# AdmixPipe v3: facilitating population structure delimitation from SNP data

**DOI:** 10.1093/bioadv/vbad168

**Published:** 2023-11-23

**Authors:** Steven M Mussmann, Marlis R Douglas, Tyler K Chafin, Michael E Douglas

**Affiliations:** Department of Biological Sciences, University of Arkansas, Fayetteville, AR 72701, United States; Abernathy Fish Technology Center, U.S. Fish and Wildlife Service, Longview, WA 98632, United States; Department of Biological Sciences, University of Arkansas, Fayetteville, AR 72701, United States; Department of Biological Sciences, University of Arkansas, Fayetteville, AR 72701, United States; Biomathematics and Statistics Scotland, Edinburgh EH49 3FD, United Kingdom; Department of Biological Sciences, University of Arkansas, Fayetteville, AR 72701, United States

## Abstract

**Summary:**

Quantifying genetic clusters (=populations) from genotypic data is a fundamental, but non-trivial task for population geneticists that is compounded by: hierarchical population structure, diverse analytical methods, and complex software dependencies. AdmixPipe v3 ameliorates many of these issues in a single bioinformatic pipeline that facilitates all facets of population structure analysis by integrating outputs generated by several popular packages (i.e. CLUMPAK, EvalAdmix). The pipeline interfaces disparate software packages to parse Admixture outputs and conduct EvalAdmix analyses in the context of multimodal population structure results identified by CLUMPAK. We further streamline these tasks by packaging AdmixPipe v3 within a Docker container to create a standardized analytical environment that allows for complex analyses to be replicated by different researchers. This also grants operating system flexibility and mitigates complex software dependencies.

**Availability and implementation:**

Source code, documentation, example files, and usage examples are freely available at https://github.com/stevemussmann/admixturePipeline. Installation is facilitated via Docker container available from https://hub.docker.com/r/mussmann/admixpipe. Usage under Windows operating systems requires the Windows Subsystem for Linux.

## 1 Introduction

Genetic population structure defined by codominant molecular markers can be readily assessed using several popular algorithms (Pritchard *et al.* 2000, Alexander and Lange 2011). While the application of these programs is a relatively simple task, the subsequent parsing and interpreting of output falls to accessory programs that determine the best models of population structure (Kopelman *et al.* 2015, Garcia-Erill and Albrechtsen 2020).

The number of genetic clusters (*K*; Pritchard *et al.* 2000) thus stems from a *post hoc* analysis, the assessment of which is confounded by intervening variables (Cullingham *et al.* 2020). Researchers often seek the ‘best’ explanation of population structure (i.e. a single *K*-estimate) using statistical parameters to parse estimates from several software packages. For example, Admixture offers cross-validation (CV) values which gauge the best *K* by minimizing prediction error among multiple programmatic iterations for different *K*-values (Alexander and Lange 2011). In contrast, the *ΔK* statistic measures rate of change from the ‘log probability of data’ among successive *K*-values (Evanno *et al.* 2005). However, erroneously low values can emerge when hierarchical population structure is encountered (Janes *et al.* 2017). Finally, the EvalAdmix algorithm calculates the correlation of residuals between actual and predicted genotypes for a given Admixture model, with an optimal *K*-value represented by correlations approximating 0 (Garcia-Erill and Albrechtsen 2020).

Thus, software packages that do not natively interface with one another are required to efficiently gauge an optimal *K* (Kopelman *et al.* 2015, Garcia-Erill and Albrechtsen 2020). CLUMPAK identifies multimodal population structure results from replicates performed in Admixture (i.e. major and minor clusters), but these are not easily parsed from the outputs of ancillary software packages. For example, EvalAdmix does not natively process multiple replicates per *K*, and the variability among CV values from Admixture replicates must be parsed following CLUMPAK evaluation. These tasks are left for individual users, with numerous liabilities subsequently emerging: individual inconsistencies in performing these necessary data conversions are one, and inadequacies in computer programming languages are the second. Both often prevent analytical repeatability (Wratten *et al.* 2021).

AdmixPipe v3 mitigates these repeatability and programming limitations by improving the original AdmixPipe (Mussmann *et al.* 2020a) to directly interface with algorithms designed to infer optimal *K*. It combines novel and updated functions for data filtering, population structure analyses, optimal *K* evaluation, and ancestry visualization into a single high-throughput analytical pipeline. The pipeline is further extended by allowing for more flexibility in user inputs. Overall, these improvements will facilitate population structure analyses and promote their replicability, particularly for non-model organisms.

## 2 Methods

### 2.1 Pipeline additions and improvements

**Figure 1.**
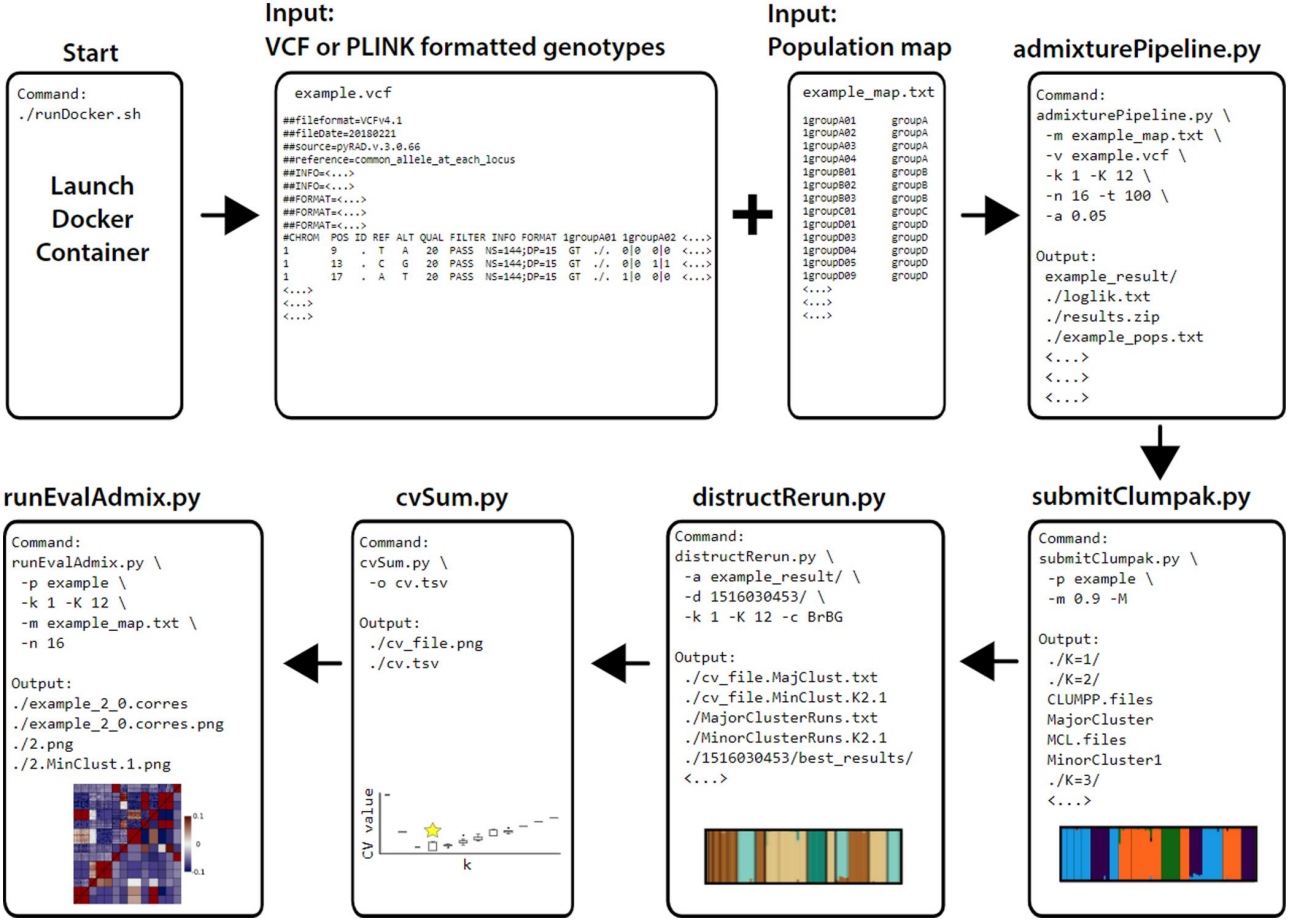
The workflow for AdmixPipe v3 requires two input files: (1) VCF or PLINK-formatted file of genotypes and (2) Tab-delimited population map. Either binary or text format can be used to input PLINK format. Both files are processed by admixturePipeline.py, which handles data filtering steps and executes Admixture. Outputs are then processed through the submitClumpak.py module to drive a local installation of CLUMPAK. Subsequently, distructRerun.py will parse CLUMPAK results and depict outputs. The cvSum.py module calculates CV values and log-likelihood values for all major and minor clusters detected by CLUMPAK. Finally, runEvalAdmix.py will evaluate fit of Admixture models to the genotype data by executing EvalAdmix for all major and minor clusters.

AdmixPipe v3 retains all functionality introduced in earlier versions (Mussmann *et al.* 2020a) but now with new features that streamline the analytical process (Fig. 1). Improvements were made in (i) inputs and filtering; (ii) CLUMPAK output parsing and plotting; (iii) testing Admixture model fit, and (iv) ease of installation.

Input requirements have been relaxed to accept greater flexibility of genotype data formats. Direct input of PLINK-formatted files [both binary (.bed) and text-based (.ped)] is now possible; however, ped files must be encoded using the—recode12 option in PLINK. Filtering of unwanted individuals has been streamlined via automatic exclusion of samples absent from the required AdmixPipe population map.

Two major improvements have been applied to the CLUMPAK output processing to facilitate direct comparison of *K*-values. First, the multiple modes (i.e. major and minor clusters) detected among Admixture replicates are now evaluated in all post-CLUMPAK components of AdmixPipe. Whereas previous pipeline versions considered only major clusters in their outputs, the CV values for minor clusters are now summarized in the box plots produced by the cvSum.py module. Second, the cvSum.py module summarizes log-likelihood values for both major and minor clusters at each *K*. These were excluded from earlier AdmixPipe versions because Admixture does not directly transmit log-likelihood values to CLUMPAK. These novel pipeline outputs more appropriately facilitate direct comparison of *K*-values, and the major/minor clusters within each *K*.

The two largest additions to AdmixPipe v3 are new modules that further streamline optimal *K* determination: (i) submitClumpak.py, which operates as a wrapper for a local installation of the CLUMPAK pipeline, thereby eliminating reliance upon the CLUMPAK webserver, and (ii) runEvalAdmix.py, which executes EvalAdmix on each replicate of each *K*-value and interfaces with CLUMPAK to inform which replicates correspond to major and minor clusters. The EvalAdmix residuals matrix is then averaged for each major and minor cluster, and R functions packaged with EvalAdmix are then used for visualization. These are called from the Python rpy2 library, thus requiring scant end-user knowledge of R. The EvalAdmix outputs provide the user with an additional measure of Admixture model fit to their input data.

Finally, a Docker container now simplifies the installation process. Direct implementation of CLUMPAK and EvalAdmix substantially increased the number of required software dependencies. The burden of manual pipeline configuration was therefore eliminated by the development of a Docker container that streamlines the installation process. All pipeline dependencies are included and configured for the end-user.

### 2.2 Test data and run parameters

We demonstrate the improvements inherent to AdmixPipe v3 by processing single nucleotide polymorphism (SNP) data for *Rhinichthys osculus* (Speckled Dace), a small minnow (Leuciscidae) with several subspecies endemic to the Death Valley region of California and Nevada, USA (Mussmann *et al.* 2020b). The dataset contains *N* = 140 individuals spanning several discrete populations; some of which have been isolated from one another for millennia. In addition to spatial population structure, temporal structure is expected in this dataset, given that separate collections for two of its localities spanned nearly 30 years. AdmixPipe v3 was executed using the original analytical parameters for this dataset (Mussmann *et al.* 2020b), with the exception that *K*-values 1–14 were evaluated. The new pipeline modules (submitClumpak.py and runEvalAdmix.py) were executed with default settings. Hierarchical population structure was explored to derive optimal *K* by applying two separate methods. First, optimal *K* was determined via lowest mean CV value calculated by Admixture. Second, EvalAdmix identified the *K*-value associated with a correlation of residuals between true and predicted genotypes best approximating zero. All pipeline commands are provided in Supplementary File S1.

## 3 Results

**Figure 2.**
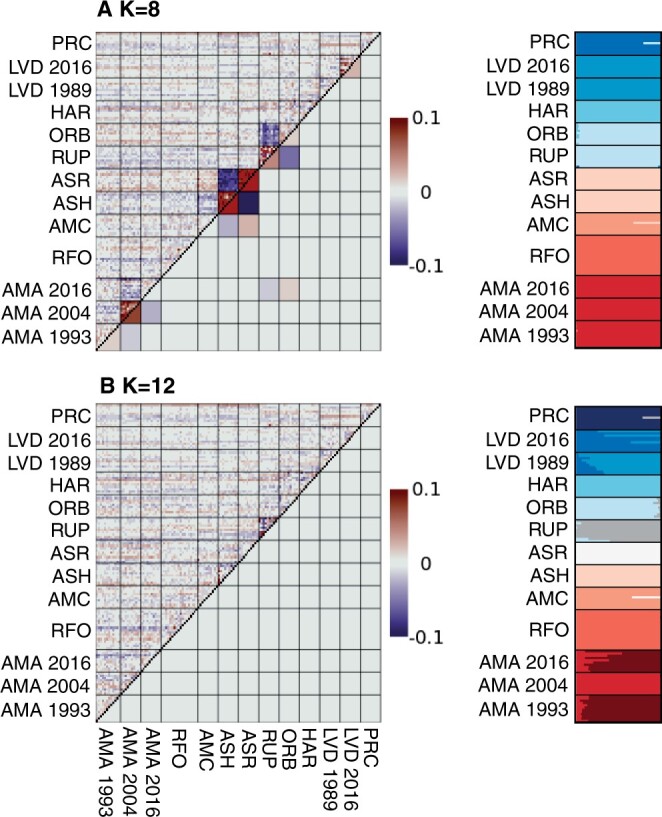
Comparison of optimal K determination from: (A) *K* = 8 Admixture-computed CV values, and (B) *K* = 12 EvalAdmix correlation of residuals. The matrices computed in EvalAdmix (left) show the correlation of residuals for actual and inferred genotypes of all individuals under different Admixture models. Bar plots (right) show the proportion of ancestry for individuals under each Admixture model.

The two methods that determined optimal *K* converged upon different population structure models. Eight genetic clusters (*K *=* *8) were favored by the CV value approach (Fig. 2A), but with log-likelihood values indicating potentially improved Admixture model fit as *K* increased. These higher *K*-values were then assessed in EvalAdmix, where the correlation of residuals between true genotypes and predicted genotypes according to the Admixture model were minimized via twelve genetic clusters (*K *=* *12) (Fig. 2B). CV and log-likelihood values for each major and minor cluster were plotted in Supplementary Figs S1 and S2.

The *K *=* *12 (EvalAdmix) model indicated more nuanced population structure relative to our original analysis (Mussmann *et al.* 2020b). Sites with temporally split collections (i.e. localities AMA and LVD) were each divided among multiple genetic clusters. Additionally, collections of subspecies groups from different sites were subdivided among genetic clusters that more accurately represent contemporary breeding populations (i.e. Ash Meadows Speckled Dace from isolated desert springs ASH and ASR, and Owens Valley Speckled Dace from localities ORB and RUP).

## 4 Conclusion

Our enhancements to AdmixPipe v3 create a path to improved repeatability of population structure analyses and facilitate the determination of optimal *K*-values via multiple methods. These improvements were verified via our test data, in which CV values illuminated an Admixture model (*K *=* *8) aligning with longer-term evolutionary processes (i.e. subspecies groups) whereas the EvalAdmix method identified contemporary population clusters (*K *=* *12) reflecting ecological processes (i.e. breeding groups). These improvements to AdmixPipe elucidated nuanced hierarchical population structure in our test data that was not detected using previous AdmixPipe versions (Mussmann *et al.* 2020b).

Our pipeline improvements streamline the export of data to accessory programs for determining optimal *K*-values and facilitate cross-reference of CLUMPAK outputs with those from other pipeline components. These features allow the user to document and report how optimal *K* was determined, explore potential hierarchical structure, assess multimodality within replicates of a given *K*, and utilize multiple methods to determine optimal *K*; thereby facilitating fulfillment of recommendations for reporting assignment test results (Janes *et al.* 2017). In closing, AdmixPipe v3 provides a standardized environment within which researchers can not only perform these necessary procedures but also report program settings that allow for a trouble-free replication of analytical effort.

## Supplementary Material

vbad168_Supplementary_Data

## Data Availability

No new genotype data were generated for this study. SNP data used for testing are available in a Dryad Digital Repository (https://doi.org/10.5061/dryad.51c59zw62). These data were derived from raw fastq files uploaded to the NCBI Sequence Read Archive (BioProject ID PRJNA598959). All Python code is available at https://github.com/stevemussmann/admixturePipeline/.
