# Supplementary material for: AdmixPipe v3: facilitating population structure delimitation from SNP data
Source: Bioinform Adv. 2023 Nov 23;3(1):vbad168. doi: 10.1093/bioadv/vbad168 (PMC10689661; doi:10.1093/bioadv/vbad168)
Supplement: vbad168_Supplementary_Data [file vbad168_supplementary_data.zip › supplementary_file_s1.docx]

#!/bin/bash

#Input Files

POPMAP="popmap.txt"

PREFIX="spd_dv"

VCF="${PREFIX}.vcf"

#admixturePipeline.py

k=1

K=14

NP=8

THIN=120

MAF=0.01

#submitClumpak.py

MCL=0.9

EMAIL="smussmann@gmail.com"

admixturePipeline.py -m $POPMAP -v $VCF -k $k -K $K -n $NP -t $THIN -a $MAF

submitClumpak.py -p $PREFIX -m $MCL -M

unzip 1668897899.zip

distructRerun.py -a ./ -d 1668897899/ -k $k -K $K

runEvalAdmix.py -p $PREFIX -k $k -K $K -m $POPMAP -n $NP

exit
