# Supplementary material for: AdmixPipe v3: facilitating population structure delimitation from SNP data
Source: Bioinform Adv. 2023 Nov 23;3(1):vbad168. doi: 10.1093/bioadv/vbad168 (PMC10689661; doi:10.1093/bioadv/vbad168)

Supplementary Figure 1. Box Plots of cross validation (CV) values corresponding to major and minor clusters detected by CLUMPAK.


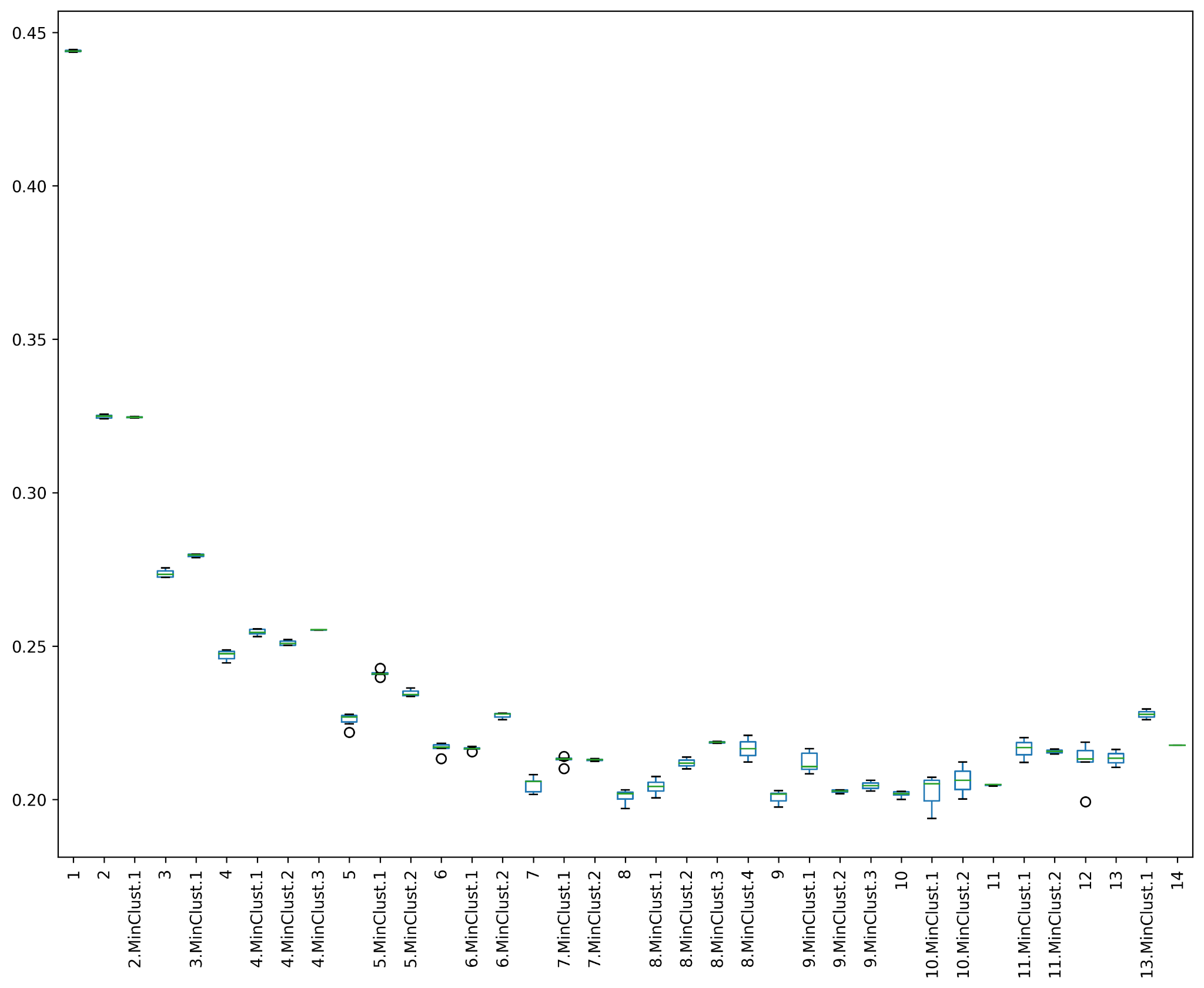


Supplementary Figure 2. Box Plots of log likelihood values corresponding to major and minor clusters detected by CLUMPAK.


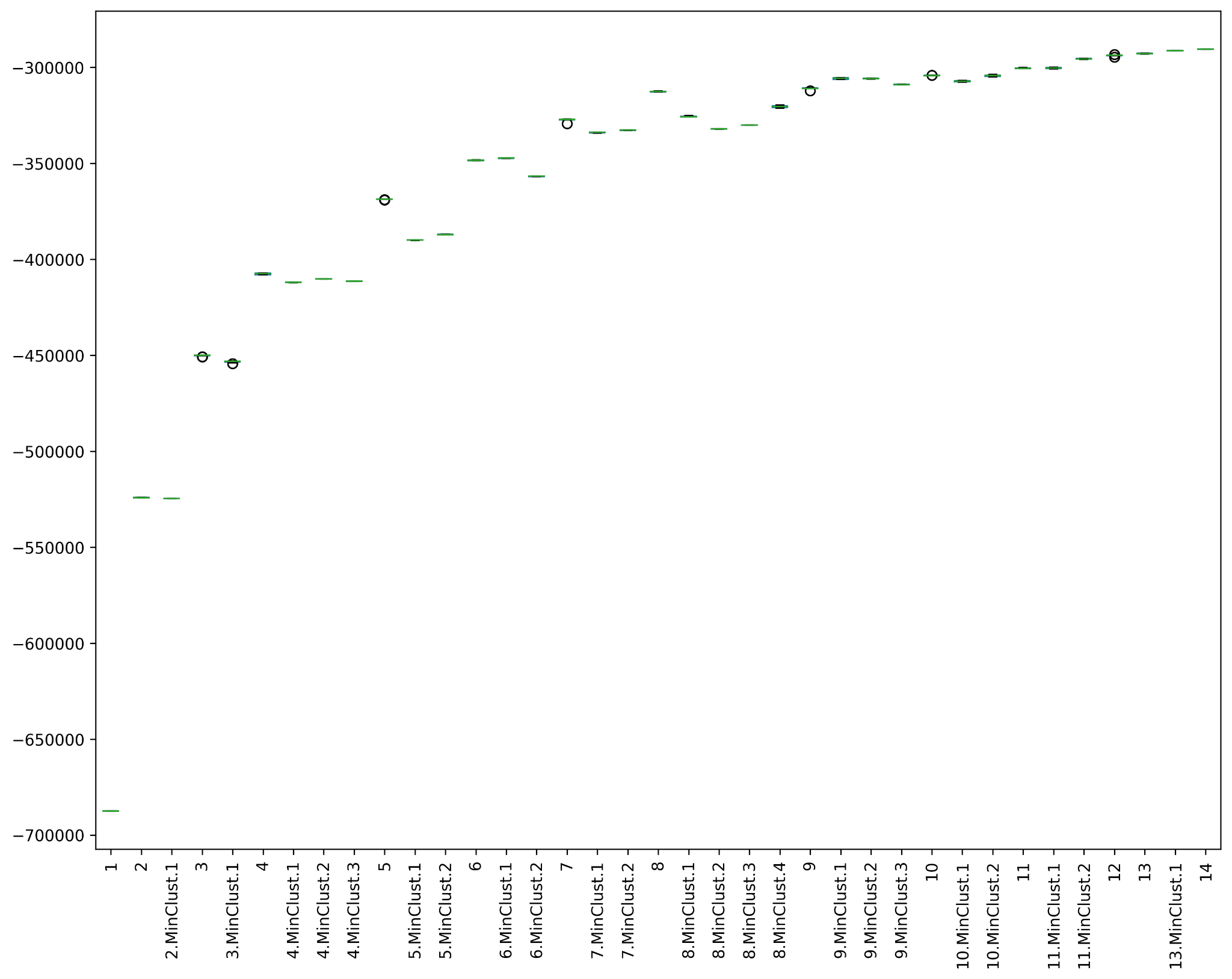

Supplement: vbad168_Supplementary_Data [file vbad168_supplementary_data.zip › supplementary_figures_1_and_2.docx]
